# Independent Microevolution Mediated by Mobile Genetic Elements of Individual Clostridium difficile Isolates from Clade 4 Revealed by Whole-Genome Sequencing

**DOI:** 10.1128/mSystems.00252-18

**Published:** 2019-03-26

**Authors:** Yuan Wu, Chen Liu, Wen-Ge Li, Jun-Li Xu, Wen-Zhu Zhang, Yi-Fei Dai, Jin-Xing Lu

**Affiliations:** a State Key Laboratory of Infectious Disease Prevention and Control, National Institute for Communicable Disease Control and Prevention, Chinese Center for Disease Control and Prevention, Beijing, China; b Collaborative Innovation Center for Diagnosis and Treatment of Infectious Diseases, Hangzhou, China; c Novogene Bioinformatics Institute, Beijing, China; University of California, San Francisco

**Keywords:** *Clostridium difficile*, antifungal resistance, horizontal gene transfer, microevolution, mobile genetic elements

## Abstract

Horizontal gene transfer of mobile genetic elements (MGEs) accounts for the mosaic genome of Clostridium difficile, leading to acquisition of new phenotypes, including drug resistance and reconstruction of the genomes. MGEs were analyzed according to the whole-genome sequences of 37 C. difficile isolates with a variety of sequence types (STs) within clade 4 from China. Great diversity was found in each transposon even within isolates with the same ST. Two novel transposons were identified in isolates ZR9 and ZR18, of which approximately one third to half of the genes showed heterogenous origins compared with the usual intestinal bacterial genes. Most importantly, *catD*, known to be harbored by Tn*4453a/b*, was replaced by *aac(6*′*) aph(2*′′*)* in isolates 2, 7, and 28. This phenomenon illustrated the frequent occurrence of gene exchanges between C. difficile and other enterobacteria with individual heterogeneity. Numerous prophages and CRISPR arrays were identified in C. difficile isolates of clade 4. Approximately 20% of spacers were located in prophage-carried CRISPR arrays, providing a new method for typing and tracing the origins of closely related isolates, as well as in-depth studies of the mechanism underlying genome remodeling. The rates of drug resistance were obviously higher than those reported previously around the world, although all isolates retained high sensitivity to vancomycin and metronidazole. The increasing number of C. difficile isolates resistant to all antibiotics tested here suggests the ease with which resistance is acquired *in vivo*. This study gives insights into the genetic mechanism of microevolution within clade 4.

**IMPORTANCE** Mobile genetic elements play a key role in the continuing evolution of Clostridium difficile, resulting in the emergence of new phenotypes for individual isolates. On the basis of whole-genome sequencing analysis, we comprehensively explored transposons, CRISPR, prophage, and genetic sites for drug resistance within clade 4 C. difficile isolates with different sequence types. Great diversity in MGEs and a high rate of multidrug resistance were found within this clade, including new transposons, Tn*4453a/b* with *aac(6*′*) aph(2*′′*)* instead of *catD*, and a relatively high rate of prophage-carried CRISPR arrays. These findings provide important new insights into the mechanism of genome remodeling within clade 4 and offer a new method for typing and tracing the origins of closely related isolates.

## Abstract (precis)

Mobile genetic elements play a key role in the continuing evolution of Clostridium difficile, resulting in the emergence of new phenotypes for individual isolates. On the basis of whole-genome sequencing analysis, we comprehensively explored transposons, CRISPR, prophage, and genetic sites for drug resistance within clade 4 C. difficile isolates with different sequence types. Great diversity in MGEs and a high rate of multidrug resistance were found within this clade, including new transposons, Tn*4453a/b* with *aac(6*′*) aph(2*′′*)* instead of *catD*, and a relatively high rate of prophage-carried CRISPR arrays. These findings provide important new insights into the mechanism of genome remodeling within clade 4 and offer a new method for typing and tracing the origins of closely related isolates.

## INTRODUCTION

Clostridium difficile, a Gram-positive, anaerobic bacterium, has emerged as the leading cause of antimicrobial and health care-associated diarrhea in humans (1). C. difficile is widespread in the environment and the gastrointestinal tracts of humans and animals (2). CD630, which was the first fully sequenced and annotated closed genome of C. difficile, comprises a large circular chromosome of 4.3 Mb, with low GC content (29.06%) (3, 4). Subsequently, thousands of whole-genome sequences (WGS) of C. difficile were published, with sizes ranging from 4.1 to 4.3 Mb (5–7). The high proportion (about 11% in strain 630) of mobile genetic elements (MGEs) contributes to the remarkable dynamic and mosaic genome of C. difficile (8). The MGEs include transposable and conjugative elements, plasmids, bacteriophages, insertion sequences (IS), clustered regularly interspersed short palindromic repeat (CRISPR) elements, group I introns, and *sigK* intervening (skin) elements (9, 10). These elements play a role in horizontal gene transfer (HGT) between distinct C. difficile isolates and between C. difficile and other intestinal pathogens, such as Enterococcus faecalis, and Enterococcus faecium (9, 11–13).

According to the multilocus sequence typing (MLST) scheme established by Griffiths et al. (14), five distinct phylogenetic lineages (clades 1 to 5) are widely recognized, and an additional clade, clade C-I, was identified, which was confirmed by WGS studies (15–17). Clade 1 is highly heterogeneous and includes many clinically significant toxigenic and nontoxigenic MLST sequence types (STs) and PCR ribotypes (RTs), such as RTs 014, 002, and 018 and STs 2, 14, and 49 (18, 19). A representative of clade 2 is hypervirulent RT027, which has caused severe outbreaks, especially in Europe and North America (20–23). Within clade 3, RT023 and STs 5 and 22 were isolated from humans in Europe (18, 24). In clade 4, RT017 (ST37) has been associated with outbreaks in Europe (25, 26) and North America (27) and is responsible for most C. difficile infections (CDIs) in Asia (28). In addition, RT017 is often clindamycin and fluoroquinolone resistant. Clade 5 contains not only RT078 but also RTs 033, 045, 066, 126, 127, 237, 280, 281, and 288 from a diverse collection of clinical, animal, and food sources worldwide and also demonstrates great heterogeneity (29, 30).

In this study, the WGS of 37 clinical C. difficile isolates from clade 4 with divergent origins in China were obtained, annotated, and analyzed. The genomic regions encoding toxins A and B (PaLoc) and the binary toxin (CdtLoc), MGEs and molecular typing (MLST and PCR ribotyping) were compared and analyzed systematically based on the WGS data. In addition, antibiotic resistance genes, putative resistance point mutation sites, and the relationship with the results of drug susceptibility tests were evaluated. These results will further our knowledge of the genetic characterization of clade 4 and further elucidate the role of MGEs in the evolution, pathogenicity, and transfer of drug resistance genes and toxin-related genes of C. difficile.

## RESULTS

### Genomic characteristics and population structure in clade 4.

**FIG 1:**
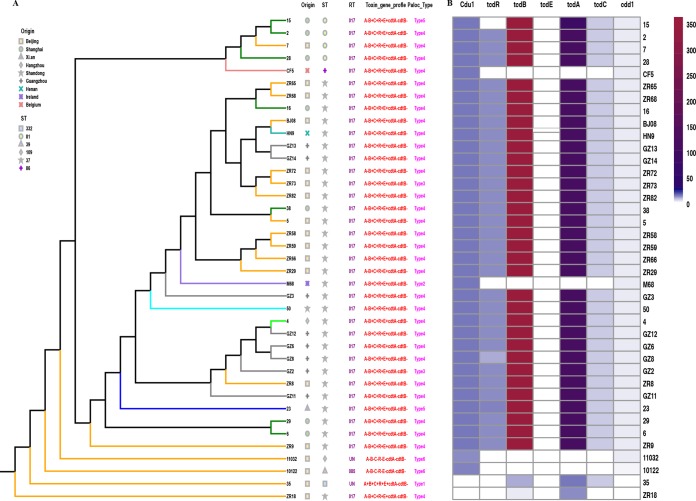
Population structure of clade 4 based on the neighbor-joining (N-J) phylogenetic tree and single nucleotide polymorphisms in the PaLoc of all tested isolates. (A) N-J tree of 37 C. difficile isolates with diverse origins and STs. (B) SNPs identified within the PaLoc of C. difficile isolates.

Details of the genome sizes of these 37 C. difficile isolates (range from 3.9 to 4.3 Mb), and other common features are summarized in Table S1 in the supplemental material. The population structure of clade 4 based on the neighbor-joining (N-J) phylogenetic tree correlated well with groups defined according to MLST, PCR ribotyping, and toxin profile but showed no obvious correlation with geographic distribution (Fig. 1). RT017 had two distinct sublineages, ST37 and ST81 (Fig. 1). Each ST cluster was highly conserved, although within the ST37 cluster, ZR18 displayed obvious distinctions compared with the rest of the ST37 isolates (Fig. 1). In the subsequent analysis, we explored the reasons for these distinctions at the genomic level. ST332 was a novel ST that we identified with a unique positive *tcdA* gene, which formed a singleton in the N-J tree (Fig. 1). Two nontoxigenic isolates (ST109 and ST39) also spread as singletons (Fig. 1).

10.1128/mSystems.00252-18.1 TABLE S1 Common genomic features of 37 C. difficile isolates. Download Table S1, PDF file, 0.06 MB. Copyright © 2019 Wu et al. 2019 Wu et al. This content is distributed under the terms of the Creative Commons Attribution 4.0 International license.

### Genetic diversity of MGEs in clade 4 (transposons, prophages, CRISPRs, and plasmids).

**FIG 2:**
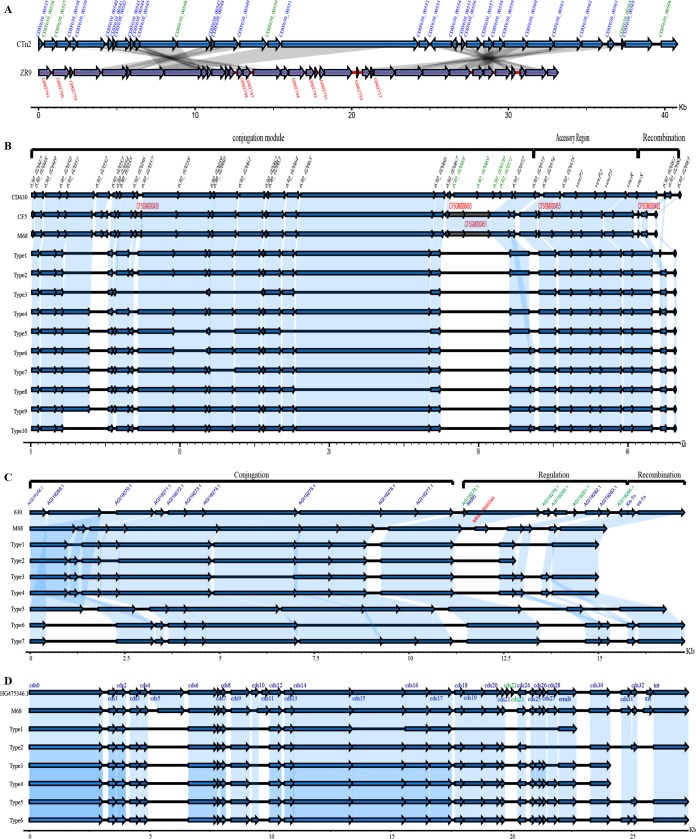
Schematic representation and comparisons of CTn*2*, CTn*5*, Tn*916*, and Tn*6194* in target isolates. Blue arrows indicate homologous genes. ORF names in red are unique in related isolates, and ORF names in green are unique to the reference isolates. Regions of homology among isolates are indicated by gray (A) and light blue shading (B to D). (A) Schematic comparisons of CTn*2* and new putative Tn in ZR9. (B to D) Schematic comparisons of different types of CTn*5* (B), Tn*916* (C), and Tn*6194* (D).

**FIG 3:**
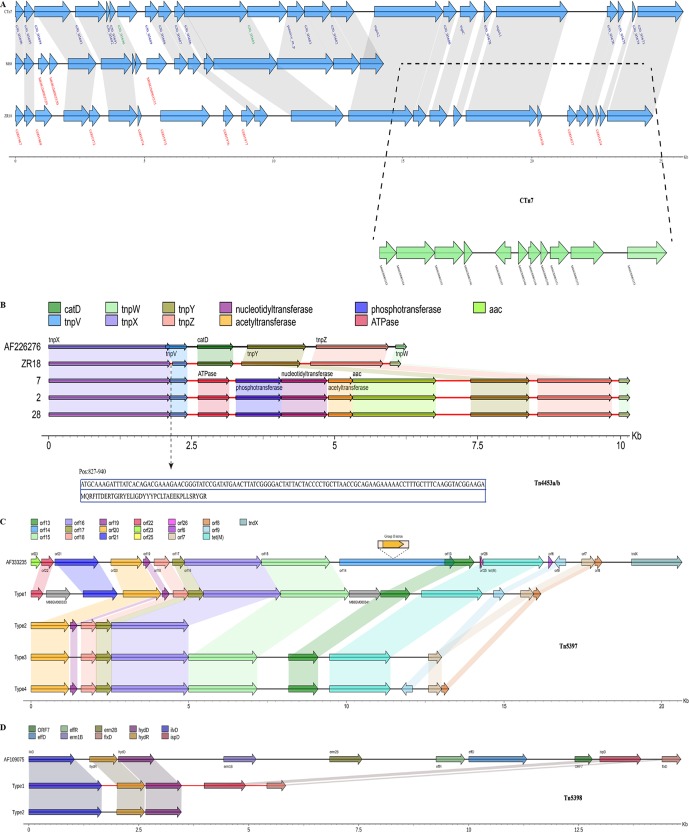
Schematic representation and comparisons of CTn*7*, Tn*4453a/b*, Tn*5397*, and Tn*5398* in target isolates. (A) For CTn*7*, blue arrows refer to homologous genes. ORF names in red are unique in related isolates, and ORF names in green are unique to reference isolates. Regions of homology among isolates are indicated by gray shading. The missing genes of M68 are displayed in regions between the dashed lines in green. For the remaining Tns, different ORFs are represented by distinct colors, and homologous regions are shown in related colors. (B) In Tn*4453a/b*, the deleted fragment (amino acids and nucleotides) in the *tnpV* gene is indicated in the dark blue box below the dashed arrow line. (C and D) Schematic comparisons of different types of Tn*5397* (C) and Tn*5398* (D).

**(i) Transposons and conjugative transposons.** Several transposons (Tns) and conjugative transposons (CTns), including CTn*5*, Tn*4453a/b*, Tn*5397*, Tn*5398*, Tn*6194*, Tn*916*, and novel putative transposons containing regions homologous with CTn*2* and CTn*7* (Table 1), were identified by comparison with reference genomes. A novel putative transposon element was discovered in isolate ZR9. This element was located in scaffold 35 from 2,689 to 3,4943 bp and contained 36 coding sequences (CDS), of which 10 were unique in this element, while the remaining 26 genes demonstrated 83.33% to 100% identity with CTn*2* (Fig. 2A). Among the 10 unique genes, G002737, G002739, G002743, G002760, and G002761 were annotated as C. difficile putative conjugative transposon protein Tn*1549*-like, CTn*2*-ORF30, CTn*2*-ORF32, CTn*5* ORF2, 18, and 19, respectively. G002741 was annotated as putative RNA methyltransferase of C. difficile E7. G002744, G002747, G002748, and G002759 were defined as hypothetical protein, bacterial regulatory helix (AraC family protein), transcriptional regulator (effector binding domain protein), and transcriptional regulator (AbrB family domain protein), respectively. In isolate ZR18, another putative novel mobile element was found, including 24 coding sequences, of which 10 genes were absent in CTn*7*, while the other genes were similar to those in CTn*7* (Fig. 3A). Large fragment insertions and deletions located in 630_03693 to 630_03688, and 630_03686 to *mgtA2* were identified in isolate ZR18 (Fig. 3A). The 10 novel genes (G001824, G001827, G001828, G001967, G001969, G001972, G001974, G001975, G001976, and G001977) in ZR18 were annotated as hypothetical protein, CTn*7*-ORF 4, 6, and conjugal transfer protein of C. difficile, and also proteins of other gut pathogens such as *Firmicutes* and Enterococcus faecium. In addition, a CTn*7*-like element was identified in reference M68, showing >85% identity with 630 CTn*7* (Fig. 3A). Two genes (M68GM003529 and M68GM003530) were inserted between 630_03695 and 630_03694. In M68, M68GM003535 replaced 630_03690 at the same site, while 630_03685 was missing (Fig. 3A). It is noteworthy that nearly half of genes in CTn*7* were lost in M68, with the addition of another 11 coding genes (Fig. 3A), encoding transcriptional regulator, ABC transporter, antimicrobial resistance gene *ermB* (M68GM003552), and *bcrA* (M68GM003544), putative truncated zeta protein, ATPase-associated proteins, and other genes originating from C. difficile and other enteric bacteria (*Erysipelotrichaceae*, E. faecalis, E. faecium, Campylobacter jejuni, *Firmicutes,* and Staphylococcus aureus).

**TABLE 1:** General description of 37 C. difficile isolates in this study

Isolate	Origin	ST	RT	Toxin gene profile	Tn^*a*^	Potential plasmid sequence	No. of prophages^*b*^
35	Beijing	332	UN	*A*^+^*B*^+^*C*^+^*R*^+^*E*^+^ *cdtA*^−^ *cdtB*^−^	Tn*5398*-like	1	7
2	Shanghai	81	017	*A*^−^*B*^+^*C*^+^*R*^+^*E*^+^ *cdtA*^−^ *cdtB*^−^	CTn*5*-like, Tn*4453a/b*, Tn*5397*-like, Tn*6194*-like	14	11
28	Shanghai	81	017	*A*^−^*B*^+^*C*^+^*R*^+^*E*^+^ *cdtA*^−^ *cdtB*^−^	CTn*5*-like, Tn*4453a/b*, Tn*5397*-like, Tn5398-like, Tn*6194*-like	13	7
7	Beijing	81	017	*A*^−^*B*^+^*C*^+^*R*^+^*E*^+^ *cdtA*^−^ *cdtB*^−^	CTn*5*-like, Tn*4453a/b*, Tn*5397*-like, Tn*5398*-like, Tn*6194*-like	14	8
15	Shanghai	81	017	*A*^−^*B*^+^*C*^+^*R*^+^*E*^+^ *cdtA*^−^ *cdtB*^−^	CTn*5*-like, Tn*5398*-like	4	9
10122	Beijing	39	085	*A*^−^*B*^−^*C*^−^*R*^−^*E*^−^ *cdtA*^−^ *cdtB*^−^	CTn*5*-like	5	5
11032	Beijing	109	UN	*A*^−^*B*^−^*C*^−^*R*^−^*E*^−^ *cdtA*^−^ *cdtB*^−^		4	3
16	Shanghai	37	017	*A*^−^*B*^+^*C*^+^*R*^+^*E*^+^ *cdtA*^−^ *cdtB*^−^	CTn*5*-like, Tn*5398*-like, Tn*6194*-like	9	8
23	Xi’an	37	017	*A*^−^*B*^+^*C*^+^*R*^+^*E*^+^ *cdtA*^−^ *cdtB*^−^	CTn*5*-like, Tn*5397*-like, Tn*5398*-like, Tn*6194*-like	5	7
29	Shanghai	37	017	*A*^−^*B*^+^*C*^+^*R*^+^*E*^+^ *cdtA*^−^ *cdtB*^−^	CTn*5*-like, Tn*5397*-like, Tn*5398*-like, Tn*6194*-like	10	6
38	Shanghai	37	017	*A*^−^*B*^+^*C*^+^*R*^+^*E*^+^ *cdtA*^−^ *cdtB*^−^	CTn*5*-like, Tn*5397*-like, Tn*6194*-like	14	8
4	Hangzhou	37	017	*A*^−^*B*^+^*C*^+^*R*^+^*E*^+^ *cdtA*^−^ *cdtB*^−^	CTn*5*-like, Tn*5397*-like, Tn*5398*-like, Tn*6194*-like	10	8
5	Beijing	37	017	*A*^−^*B*^+^*C*^+^*R*^+^*E*^+^ *cdtA*^−^ *cdtB*^−^	CTn*5*-like, Tn*5397*-like, Tn*5398*-like, Tn*6194*-like	13	9
50	Shandong	37	017	*A*^−^*B*^+^*C*^+^*R*^+^*E*^+^ *cdtA*^−^ *cdtB*^−^	CTn*5*-like, Tn*5398*-like	1	5
6	Shanghai	37	017	*A*^−^*B*^+^*C*^+^*R*^+^*E*^+^ *cdtA*^−^ *cdtB*^−^	CTn*5*-like, Tn*5397*-like, Tn*5398*-like, Tn*6194*-like	10	7
BJ08	Beijing	37	017	*A*^−^*B*^+^*C*^+^*R*^+^*E*^+^ *cdtA*^−^ *cdtB*^−^	CTn*5*-like, Tn*5397*-like, Tn*5398*-like	9	7
GZ2	Guangzhou	37	017	*A*^−^*B*^+^*C*^+^*R*^+^*E*^+^ *cdtA*^−^ *cdtB*^−^	CTn*5*-like, Tn*5397*-like, Tn*5398*-like, Tn*6194*-like	10	7
GZ3	Guangzhou	37	017	*A*^−^*B*^+^*C*^+^*R*^+^*E*^+^ *cdtA*^−^ *cdtB*^−^	CTn*5*-like, Tn*5397*-like, Tn*6194*-like	10	8
GZ6	Guangzhou	37	017	*A*^−^*B*^+^*C*^+^*R*^+^*E*^+^ *cdtA*^−^ *cdtB*^−^	CTn*5*-like, Tn*5397*-like, Tn*5398*-like, Tn*6194*-like	10	8
GZ8	Guangzhou	37	017	*A*^−^*B*^+^*C*^+^*R*^+^*E*^+^ *cdtA*^−^ *cdtB*^−^	CTn*5*-like, Tn*5397*-like	10	9
GZ11	Guangzhou	37	017	*A*^−^*B*^+^*C*^+^*R*^+^*E*^+^ *cdtA*^−^ *cdtB*^−^	CTn*5*-like, Tn*5397*-like, Tn*6194*-like	11	8
GZ12	Guangzhou	37	017	*A*^−^*B*^+^*C*^+^*R*^+^*E*^+^ *cdtA*^−^ *cdtB*^−^	CTn*5*-like, Tn*5397*-like, Tn*6194*-like	10	7
GZ13	Guangzhou	37	017	*A*^−^*B*^+^*C*^+^*R*^+^*E*^+^ *cdtA*^−^ *cdtB*^−^	CTn*5*-like, Tn*5397*-like, Tn*5398*-like, Tn*6194*-like	13	9
GZ14	Guangzhou	37	017	*A*^−^*B*^+^*C*^+^*R*^+^*E*^+^ *cdtA*^−^ *cdtB*^−^	CTn*5*-like, Tn*5397*-like, Tn*5398*-like, Tn*6194*-like	13	11
ZR58	Beijing	37	017	*A*^−^*B*^+^*C*^+^*R*^+^*E*^+^ *cdtA*^−^ *cdtB*^−^	CTn*5*-like, Tn*5397*-like, Tn*6194*-like	14	11
ZR59	Beijing	37	017	*A*^−^*B*^+^*C*^+^*R*^+^*E*^+^ *cdtA*^−^ *cdtB*^−^	CTn*5*-like, Tn*5397*-like, Tn*6194*-like	14	13
ZR65	Beijing	37	017	*A*^−^*B*^+^*C*^+^*R*^+^*E*^+^ *cdtA*^−^ *cdtB*^−^	CTn*5*-like, Tn*5398*-like	4	8
ZR66	Beijing	37	017	*A*^−^*B*^+^*C*^+^*R*^+^*E*^+^ *cdtA*^−^ *cdtB*^−^	CTn*5*-like, Tn*5397*-like, Tn*5398*-like, Tn*6194*-like	14	13
ZR68	Beijing	37	017	*A*^−^*B*^+^*C*^+^*R*^+^*E*^+^ *cdtA*^−^ *cdtB*^−^	CTn*5*-like, Tn*5398*-like	5	8
ZR72	Beijing	37	017	*A*^−^*B*^+^*C*^+^*R*^+^*E*^+^ *cdtA*^−^ *cdtB*^−^	CTn*5*-like, Tn*5397*-like, Tn*5398*-like	9	8
ZR73	Beijing	37	017	*A*^−^*B*^+^*C*^+^*R*^+^*E*^+^ *cdtA*^−^ *cdtB*^−^	CTn*5*-like, Tn*5397*-like, Tn*5398*-like	8	10
ZR82	Beijing	37	017	*A*^-^*B*^+^*C*^+^*R*^+^*E*^+^*cdtA*^-^*cdtB*^-^	CTn*5*-like, Tn*5397*-like, Tn*5398*-like	8	7
ZR29	Beijing	37	017	*A*^-^*B*^+^*C*^+^*R*^+^*E*^+^ *cdtA*^−^ *cdtB*^-^	CTn*5*-like, Tn*5397*-like, Tn*5398*-like, Tn*6194*-like	14	9
ZR18	Beijing	37	017	*A*^−^*B*^+^*C*^+^*R*^+^*E*^+^ *cdtA*^−^ *cdtB*^−^	CTn*5*-like, npe-CTn*7*, Tn*4453a/b*, Tn5397-like	7	5
ZR8	Beijing	37	017	*A*^−^*B*^+^*C*^+^*R*^+^*E*^+^ *cdtA*^−^ *cdtB*^−^	CTn*5*-like, Tn*5397*-like, Tn*5398*-like, Tn*6194*-like	10	6
ZR9	Beijing	37	017	*A*^−^*B*^+^*C*^+^*R*^+^*E*^+^ *cdtA*^−^ *cdtB*^−^	npe-CTn*2*, Tn*5397*-like, Tn*6194*-like	9	6
HN9	Henan	37	017	*A*^−^*B*^+^*C*^+^*R*^+^*E*^+^ *cdtA*^−^ *cdtB*^−^	CTn*5*-like, Tn*5397*-like, Tn*5398*-like	9	8
CD630		54	012	*A*^+^*B*^+^*C*^+^*R*^+^*E*^+^ *cdtA*^−^ *cdtB*^−^			
M68	Ireland	37	017	*A*^−^*B*^+^*C*^+^*R*^+^*E*^+^ *cdtA*^−^ *cdtB*^−^	CTn*5*-like, CTn*7*-like, Tn*5397*-like		
CF5	Belgium	86	017	*A*^−^*B*^+^*C*^+^*R*^+^*E*^+^ *cdtA*^−^ *cdtB*^−^	CTn*5*-like		

a npe, novel putative element.

b Total number of incomplete, intact, and questionable prophage.

CTn*5*-like transposons, belonging to the Tn*1549* family, were detected widely in the tested C. difficile isolates of clade 4, with the exception of isolate 35 and 11032 (Fig. 2B). Ten types were divided according to gene composition as follows (Fig. 2B): type 1 (HN9), type 2 (BJ08), type 3 (15), type 4 (GZ8), type 5 (ZR18), type 6 (GZ3), type 7 (29), type 8 (2, 28, 5, 7, ZR8, ZR9), type 9 (23, 38, 4, 50, 6, GZ11, GZ12, GZ2, GZ6, ZR29, ZR58, ZR59, Z566, 10122), and type 10 (16, GZ13, GZ14, ZR65, ZR68, ZR72, ZR73, ZR82). Two common major deletions in the conjugative region were observed in these 10 types, one being a deletion of 630_02052, and the other one located between 630_02066 and 630_02072 (Fig. 2B). Especially for types 3, 5, and 8, other large fragment deletions were also discovered in the conjugative region (Fig. 2B).

Tn*4453a/b* was found with typical gene organization in isolates ZR18, 2, 7, and 28 (Fig. 3B). The composition and location of genes in ZR18 was identical to that in reference Tn*4453a/b*, with the exception of a −114-bp deletion in *tnpv* (Fig. 3B). The key feature of this element was the presence of *catD*, which mediates resistance to chloramphenicol. Interestingly, *catD* was absent in isolates 2, 7, and 28 and replaced by five new genes: aminoglycoside acetyltransferase (*aac*), three transferases (phosphotransferase, nucleotidyltransferase, and acetyltransferase) and an ATPase (Fig. 3B). The *aac* gene, which confers resistance to aminoglycoside antibiotics, such as gentamicin, tobramycin, and netilmicin has been widely reported in *Enterococcus* and *Enterobacteria*, and can be transferred between Gram-positive and Gram-negative bacteria by transposons (Tn*5281*, Tn*4001*, and IS256) or plasmids (31). BLAST analysis revealed that the DNA sequences of *aac* in isolates 2, 7, and 28 showed 100% coverage and identity with *aac(6*′*) aph(2*′′*)* in C. jejuni. The total length of the gene was 1,455 bp, encoding a bifunctional aminoglycoside *N*-acetyltransferase and aminoglycoside phosphotransferase, which is one of the aminoglycoside-modified enzymes (AMEs), playing critical role in high-level gentamicin-resistant (HLGR) of *Enterococcus* (32).

Tn*5397*, also known as CTn*3* in CD630, was recognized in most isolates in this study and was classified into four types as follows: type 1, ZR18 and M68; type 2, ZR73 and 5; type 3, BJ08, GZ8, and HN9; type 4, ZR82, ZR9, 2, 23, 28, 29, 38, 4, 6, 7, GZ11, GZ12, GZ13, GZ14, GZ2, GZ3, GZ6, ZR29, ZR58, ZR59, ZR66, ZR72, and ZR8 (Table 1). The *tndX* gene, encoding a large serine recombinase, which is essential for insertion/excision of Tn*5397*, was absent in all types (Fig. 3C). Furthermore, a group II intron within orf14 was also deleted (Fig. 3C). Significantly, in type 2, Tn*5397* was missing some of the downstream genes, including an antimicrobial resistance gene *tetM*, compared with the reference Tn*5397* (Fig. 3C). Type 1 displayed the most similar gene composition and structure compared with the reference Tn*5397*, although two new genes were inserted (Fig. 3C).

Tn*5398*, first reported in strain 630, contains *erm 1B* and *erm 2B*, and can be transferred between C. difficile isolates, or between C. difficile and S. aureus or Bacillus subtilis (33). Two types of Tn*5398*-like elements were identified here, in which *erm 1B* and *erm 2B* genes were absent (Fig. 3D). Type 1 consisted of 35, ZR73, ZR82, 16, 23, 28, 29, 4, 5, 50, 6, 7, BJ08, GZ13, GZ14, GZ2, GZ6, HN9, ZR29, ZR65, ZR66, ZR68, ZR72, and ZR8, while type 2 was represented only by isolate 15 (Table 1).

Tn*916* is a major family transposon reported in C. difficile and carries the *tetM* resistance gene. The Tn*916* element of these tested isolates were subdivided into multiple types as follows: type 1, GZ8; type 2, ZR18; type 3, ZR73; type 4, 23, 29, 4, 6, GZ11, GZ12, GZ13, GZ14, GZ2, GZ3, GZ6, ZR72, ZR8, ZR82, and ZR9; type 5, BJ08 and HN9; type 6, 5; type 7, 2, 28, 38, 7, ZR29, ZR58, ZR59, and ZR66 (Table 1). The *tetM* resistance and *int* gene (responsible for Tn insertion), which are responsible for insertion, appeared in all types (Fig. 2C). With the exception of type 6 with deletions in three large regions, deletions occurred in a single area of the regulation region in all the other types (Fig. 2C). Intact *xis* and *int* genes were retained in types 3, 4, 6, and 7. The conjugative region was highly homologous within most types, except for types 3, 6, and 7, which had large fragment deletions (Fig. 2C). Isolate ZR18 carried no typical *xis* and *int* genes of Tn*916*, while BJ08, GZ8, and HN9 contain *int* without *xis*.

The representative gene cluster of Tn*6194* carrying *ermB* of strain CII7 (GenBank accession no. HG475346) was used as a reference in this study. Tn*6194* has a conjugation region that is closely related to that of Tn*916* but contains an accessory region that is related to Tn*5398*. M68 demonstrated high-level homology with only the absence of cds22 and cds 23, and indels within cds5 and cds 11, while intact *xis* and *int* genes were retained for excision and insertion (Fig. 2D). The following six types were defined here: type 1, 16, 2, 28, 38, 5, 7, GZ13, and GZ14; type 2, 23; type 3, ZR66; type 4, ZR29, ZR58, and ZR59; type 5, 29, 4, 6, GZ11, GZ12, GZ2, GZ6, ZR8, and ZR9; type 6, GZ3 (Table 1).The *ermB* gene was present in all types except type 2 (Fig. 2D). The *xis* gene, which is involved in the excision of Tn*916* from the donated strain were deleted in all types (Fig. 2D).The *int* gene, which plays a key role in the integration of Tn*916* into the recipient isolate, appeared in types 2, 5, and 6 only (Fig. 2D).

**FIG 4:**
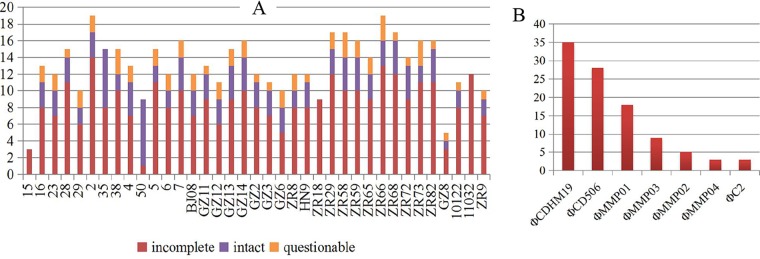
Prophage analysis of 37 C. difficile isolates from clade 4. (A) Details of the prophages in each isolate. (B) The most predominant prophages identified in clade 4. Isolates or prophages are shown on the *x* axes, and the number of prophages is shown on the *y* axes.

**(ii) Prophages, plasmids, and CRISPR.** Prophages were identified in various amounts in all tested isolates (Table S2). In total, 497 prophages were predicted in this study. Isolates 2 and ZR66 contained the highest number of prophages (19 prophages), while isolate 15 had the fewest (3 prophages) (Table S2 and Fig. 4A). A total of 115 intact prophages was confirmed among in all the isolates, except ZR18, 15, and 11032 (Fig. 4A). Among the intact prophages, the number of ΦCDHM19, ΦCD506, ΦMMP01, ΦMMP03, ΦMMP02, ΦMMP04, and ΦC2 was more than three per isolate (Fig. 4B). ΦCDHM19 and ΦCD506 represented more than half (54.78%) of all intact prophages (Fig. 4B). All of these prophages belonged to the *Myoviridae* family, and some were induced during C. difficile infection (34). Most prophages identified here were homologous to known phages reported in C. difficile, although a few were similar to other bacterial phages, such as C. jejuni, S. aureus, *Enterobacteria*, *Bacillus*, and other unusual bacteria, such as *Prochlorococcus*, *Gordonia,* and *Sphingomonas* (Table S2). Isolates 50 and 35 carried the highest number of intact prophages, 8 and 7, respectively (Table S2 and Fig. 4A). The P-SSM2-like phage of *Prochlorococcus* found in isolate 50 showed the highest number of proteins similar to those in the region (Table S2), while the ΦC2-like phage in isolate 35 displayed a high number of “hit_genes_count” in bracket (8 out of 9) with ΦC2 (Table S2). The prophages found within the ST 37 and ST 81 isolates were diverse, although ΦCD506 was predominant. No obvious correlation was found between prophage type and STs. Future studies will focus on the role of these elements in the C. difficile evolution and infection.

10.1128/mSystems.00252-18.2 TABLE S2 Detailed information on predicted prophages within 37 C. difficile isolates. Download Table S2, XLSX file, 0.05 MB. Copyright © 2019 Wu et al. 2019 Wu et al. This content is distributed under the terms of the Creative Commons Attribution 4.0 International license.

A total of 464 CRISPR arrays, composed of different copies of direct repeat (DR) sequences, and separated by unique spacers were identified in the tested isolates from clade 4 (Table S3). The number in each isolate varied from 5 to 19, and the length ranged from 60 to 1,608 bp (Table S3). In total, 90 of the CRISPR arrays displayed homology with prophages (Table S3). Isolates 10122, 11032, 29, and GZ8 harbored prophages without homologous CRISPR sequences (Table S3).

10.1128/mSystems.00252-18.3 TABLE S3 CRISPR arrays identified within 37 C. difficile isolates. Download Table S3, XLSX file, 0.04 MB. Copyright © 2019 Wu et al. 2019 Wu et al. This content is distributed under the terms of the Creative Commons Attribution 4.0 International license.

Sixteen potential plasmid sequences with almost 100% coverage were identified among the 37 isolates (Table S4) and were found to contain important antimicrobial resistance genes (*aadE*, *aad9*, *ermB*, *tetM*, *tetS*, and *tetO*) (Table S4).

10.1128/mSystems.00252-18.4 TABLE S4 Potential plasmid sequences of 37 C. difficile isolates. Download Table S4, PDF file, 0.08 MB. Copyright © 2019 Wu et al. 2019 Wu et al. This content is distributed under the terms of the Creative Commons Attribution 4.0 International license.

### Distinctions of PaLoc and CdtLoc regions in clade 4.

**FIG 5:**
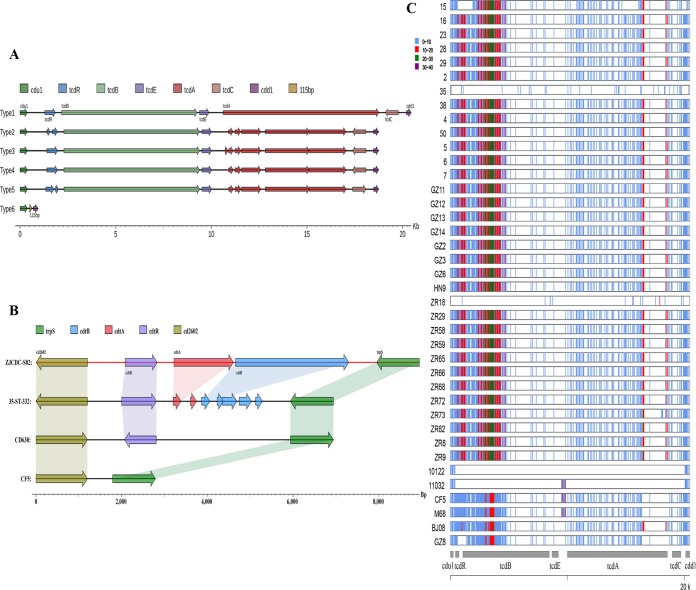
PaLoc and CdtLoc analysis of 37 C. difficile isolates from clade 4. (A) Several types were divided based on gene composition of PaLoc. (B) Gene composition of CdtLoc within clade 4. (C) SNPs distributed within PaLoc of all 37 C. difficile isolates from clade 4.

Three types of toxin gene profiles in clade 4 were covered in this study: A^+^B^+^ (ST332), A^−^B^+^ (ST81 and ST37), and A^−^B^−^ (ST39 and ST109) (Table 1). All isolates in this study were binary toxin negative (Table 1). Based on gene formation, six types of PaLoc were classified in this study (Fig. 5A). Type 1 (ST332) displayed intact gene composition and sequences, while all genes were lost with the exception of 115 bp remaining in type 6 (ST39 and ST109) (Fig. 5A). The other four types lack a complete and continuing *tcdA* gene, attributed to insertions and single nucleotide polymorphisms (SNPs), which led to termination of *tcdA* gene translation (Fig. 5A). Compared with CD630, the SNPs of genes located within PaLoc regions are shown for each isolate in Fig. 5B. In general, diversity within PaLoc regions correlated with its ST type, with some exceptions (Fig. 5B). For example, isolate 15 within ST81 and ZR18 within ST37 showed distinct SNP distribution (Fig. 5B). The *tcdB* gene demonstrated great heterogeneity, followed by *tcdA*. With the exception of isolate 35, all tested isolates lacked the CdtLoc region (Fig. 5C). Isolate 35 contained a complete *cdtR* gene and fragments of *cdtA* and *cdtB* genes (Fig. 5C).

### Antimicrobial resistance gene and related antimicrobial susceptibility.

**FIG 6:**
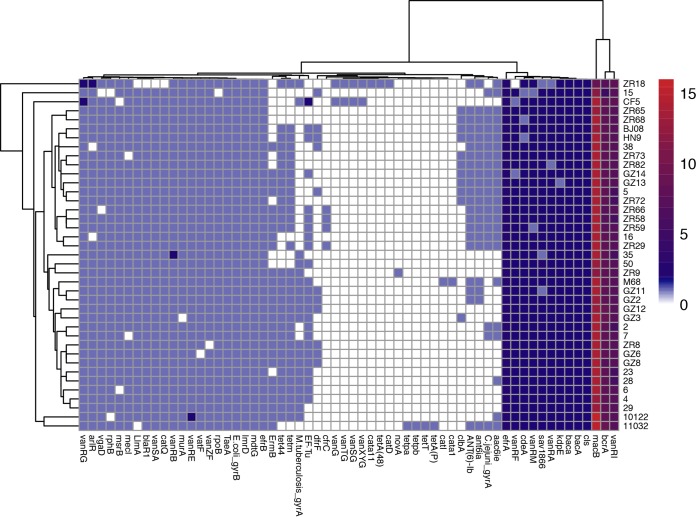
Antibiotic resistance genes of 37 C. difficile isolates predicted through comparison with the CARD and ARDB databases. The vertical coordinates refer to 37 C. difficile isolates, while the horizontal coordinates refer to the antibiotic-resistant genes identified.

In this study, 60 antibiotic resistance genes were identified by comparison with the ARDB and CARD databases (35, 36). These antibiotic resistance genes belonged to the following antimicrobial classes: fluoroquinolones, β-lactam antibiotics, macrolides, aminocoumarin, rifampin, glycopeptides, tetracycline, macrolide-lincosamide-streptogramin (MLS), chloramphenicol, trimethoprim, streptomycin, aminoglycoside, bacitracin, and lipopeptide (Fig. 6). Most isolates carried approximately 13 to 16 copies of the *macB* gene, except for ZR18 carrying 9 copies. The next highest numbers of copies were for the *vanRI* and *bcrA* genes (Fig. 6). Remarkably, some isolates carried a unique antibiotic resistance gene cassette, for example, ZR18 and CF5 contained a unique antibiotic resistance gene cluster of approximately 6 kb comprising *vanXYG*, *vanSG*, *vanTG*, and *vanG*, which were integrated as glycopeptide resistance gene (Fig. 6). Additionally, ZR18 also carried *catD* (carried by Tn*4453a/b*), and *cata11*, which are related to chloramphenicol resistance, and *tetA(48)* (Fig. 6). No functional vancomycin resistance operon was identified here. Furthermore, isolate 11032 contained unique *tetA*(P), *tetT*, *tetpb*, and *tetpa* genes (Fig. 6). In addition, ZR9 carried a unique *novA* gene, and M68 had unique *cata1* and *catl* genes (Fig. 6).

According to the Etest results, all isolates except for 10122 and 35 were MDR strains, maintaining a resistance rate as high as 97.30%. Details are summarized in Table 2. Almost all MDR strains displayed high levels of drug resistance (Table 2) with quinolone exhibiting 100% resistance to CIF, followed by LVX (97.30%) and MXF (56.76%) (drug abbreviations in “Antimicrobial susceptibility tests” in Materials and Methods) (Table 2). The quinolone resistance is due to a point mutation and substitution within *gyrA* and/or *gyrB*. Clade 4 C. difficile retained high-level resistance (91.89%) to ERY compared with the other isolates of other clades reported in Table 2. The *ermB* gene, encoding an antibiotic target-modifying enzyme, is related to MLS resistance. The *ermB* gene was detected in 25 of 34 ERY-resistant isolates (73.53%). ERY resistance is attributed to methylation of position 2058 in 23S rRNA; however, the exact mutation in ERY-resistant C. difficile isolates remains to be identified in the future. In addition, high copy numbers of the macrolide resistance *macB* gene, encoding a macrolide ABC transporter protein, were identified in all the tested isolates (Fig. 6). The rate of resistance to CLI was 89.19%, with a high level of resistance detected among the isolates. The rate of resistance to CHL was lower (29.73%) and related to *cata11* and *cata1*, which were identified only in isolates ZR18 and M68, respectively, indicating the existence of other mechanisms of CHL resistance. It is worth mentioning that *catD*, normally carried by *Tn4453a/b* and also responsible for CHL resistance (Fig. 3), was present in isolate ZR18, which showed an intermediate level of CHL resistance (MIC = 64 μg/ml). However, *catD* was replaced by another five genes in isolates 7, 2, and 28, which were CHL susceptible. The five new replacement genes included *aac(6*′*) aph(2*′*)*, which is correlated significantly with HLGR and is responsible for aminoglycoside resistance, leading to gentamicin and amikacin resistance in isolates 7, 2, and 28. The rates of MEM (51.35%) and RIF (48.65%) resistance were similar among the clade 4 C. difficile isolates. All isolates resistant to CHL and RIF were ST37 (Table 2). The *rpoB* and *rphB* genes with several mutations related to RIF resistance were detected in almost all isolates. The mechanism underlying this resistance involves substitution of several nucleotides in the *rpoB* gene. None of the clade 4 isolates were resistant to VAN or MET, although VAN-related genes or gene cassettes were identified.

**TABLE 2:** Antimicrobial resistance results and correlation with ST types and antibiotic resistance genes

Drug (concn range [μg/ml])	No. of resistant isolates (%)^*a*^	ST^*b*^	No. of genes identified^*b*^	MIC (μg/ml)^*b*^
MXF (0.002–32)	21 (56.76)	ST81 (4), ST109 (1), ST37 (16)	*E*. *coli gyrB* (37), *M. tuberculosis gyrA* (18), C. jejuni *gyrA* (20)	≥32 (21)

LVX (0.002–32)	36 (97.30)	ST332 (1), ST39 (1), ST109 (1), ST81 (4), ST37 (29)	As above	≥32 (35)
				≥8 < 32 (1)

CIF (0.002-32)	37 (100)	ST332 (1), ST39 (1), ST109 (1), ST81 (4), ST37 (30)	As above	≥32 (36)
				≥8 < 32 (1)

CLI (0.016–256)	33 (89.19)	ST81 (4), ST109 (1), ST37 (28)	*ermB* (25), *cfrC* (4), *clbA* (11)	≥256 (32)
				≥8 < 256 (1)

TET (0.016–256)	13 (35.14)	ST81 (2), ST37 (11)	*tetA*(*P*), *tetT*, *tetpb*, *tetpa* were only in isolate 11032; *tetA*(48)^*c*^ was only in ZR18; *tetM* (31), *tet44* (30)	≥16 < 32 (13)

ERY (0.016–256)	34 (91.89)	ST81 (4), ST39 (1), ST109 (1), ST37 (28)	*ermB* (25), *macB* (37), *cfrC* (4)	≥256 (32)
				≥128 < 256 (2)
				≥8 < 128 (1)

CHL (0.016–256)	11 (29.73)	ST37 (11)	*cata11* was only in ZR18; *cata1* was only in M68	≥256 (8)
				≥128 < 256 (1)
				≥32 < 128 (2)

MEM (0.002–32)	19 (51.35)	ST81 (1), ST109 (1), ST37 (17)	*blaR1* (36), *mecI* (35)	≥32 (15)
				≥16 < 32 (4)

RIF (0.002–32)	18 (48.65)	ST37 (18)	*rpoB* (36), *rphB* (34)	≥32 (18)

VAN (0.016–256)	0	0	*vanXYG*-*vanSG*-*vanTG*-*vanG* gene cluster was only in ZR18; *vanRI* (37), *vanRM* (37), *vanRA* (37), *vanRF* (36), *vanZF* (36), *vanRE* (37), *vanRB* (37), *vanSA* (36), *vanRG* (37), *arlR* (34)	≤0.5

MET (0.016–256)	0	0	None	≤1

a Values in parentheses show the percentage of isolates resistant to a single drug.

b Values in parentheses show the number of isolates with the ST, gene, or MIC.

c Gene name.

### Genome-wide association analysis of resistance genes.

**FIG 7:**
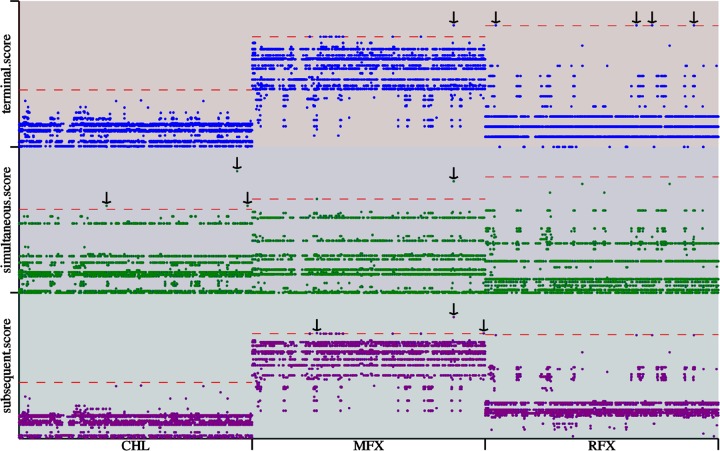
The whole-genome sequence analysis of antimicrobial resistance among 37 C. difficile isolates using treeWAS software. The vertical coordinates represent three different calculating methods, which are displayed in three distinct colors. The horizontal coordinates refer to three drugs. The significant SNPs related to each drug are highlighted by black arrows.

**FIG 8:**
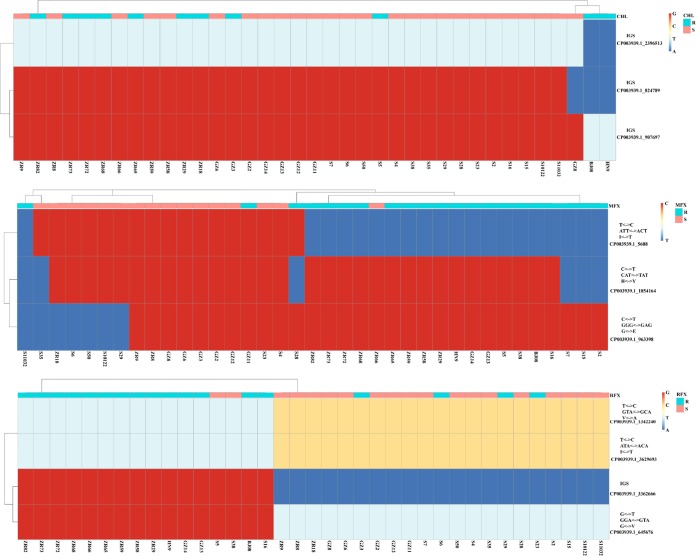
Heatmap of point mutations associated with drug resistance among all 37 C. difficile isolates. The drug, nucleotide, codon, amino acid, and gene ID are shown on the right vertical axes. The name of each isolate is shown on the horizontal axes. Nucleotides A, T, C, G are represented by dark blue, light blue, yellow, and red, respectively. R, resistance; S, susceptible.

According to our analysis, antimicrobial susceptibility does not correlate well with genotypes. To explore other potential sites responsible for drug resistance, we performed genome-wide association study (GWAS) analysis. Significantly related SNPs were detected in relation to resistance to CHL, MFX, and RIF (Fig. 7). Three significant SNPs associated with CHL resistance were located within an intergenic genetic region (IGS) (Fig. 8), which may play a role in methylation and regulation of cell function. Three remarkable nonsynonymous SNPs associated with MFX resistance (Fig. 7) were detected in genes encoding inosine-uridine-preferring nucleoside hydrolase family protein and the ABC transporter and in the *gyrA* gene, which are reported to be associated with quinolone resistance, and were detected using all three methods (Fig. 7). All MFX-resistant isolates except for GZ11 and 28 displayed the point mutation (Thr→IIe) in *gyrA* compared with susceptible isolates (Fig. 8). In addition, only one MFX-susceptible isolate ZR66 carried the Thr→IIe mutation in *gyrA* (Fig. 8). As shown in Fig. 7, four significant SNPs significantly related to RIF resistance were identified as following: a gene (*dacC* or *dacD*) encoding D-alanyl-D-alanine carboxypeptidase protein, also known as penicillin-binding protein (WP_022616436.1), gene *merR* encoding the transcriptional regulator (WP_022616524.1), a gene involved in replication, recombination, and repair (WP_022616400.1), and a gene in an IGS region (Fig. 8). Most of the RIF-resistant isolates carried the point mutation Ala→Val, Thr→IIe, and Val→Gly in these genes, respectively, with the exception of isolates GZ3, 6, 29, and 23 (Fig. 8). The RIF-susceptible isolates 5 and 38 carried the same mutations (Fig. 8).

## DISCUSSION

Due to rapid progress in WGS techniques, the global population structure of C. difficile is defined as 5 main clades (clades 1 to 5). ST37 (RT017), which is a well-known representative of clade 4, has caused outbreaks in Europe and in the United States, and is documented as the major type associated with CDI in Asia (8). In addition to ST37, clade 4 contained some diversity, such as ST332 (A^+^B^+^), ST39 and ST109 (A^−^B^−^), ST81 (A^−^B^+^), and ST86 (A^−^B^+^). ST332 is a novel type previously identified by our group (37). In this study, we analyzed and compared the whole-genome sequences of 37 clinical C. difficile isolates from clade 4. M68 and CF5 were included as isolates of clade 4, and CD630 was used as reference. We further explored the presence of Tn/CTns, prophages, and CRISPRs and compared their characteristics among the isolates in this clade. The antimicrobial phenotypes of these isolates were determined, and possible antibiotic resistance-related genes were identified. In this study, we aimed to clarify the genetic heterogeneity and microevolution within clade 4 to provide potential explanations for the mechanisms of pathogenesis and antibiotic resistance of C. difficile.

The basic features, including GC% and genome size, of the clade 4 isolates from China investigated in this study were similar to those have been reported previously (3). The C. difficile genome is highly mosaic and dynamic in structure because of a high proportion of MGEs. CTns, also known as integrated conjugative elements (ICEs), are capable of excision, transfer, and integration into the chromosome or plasmids, resulting in the formation of a circular intermediate (12). Within C. difficile, there are two main families of CTns, known as the Tn*916*- and the Tn*1549*-like elements. Tn*916* is an 18-kb conjugative transposon that encodes resistance to tetracycline and minocycline via the ribosomal protection protein, Tet (M), and contains 24 potential ORFs. Tn*1549*, a 34-kb vancomycin resistance element controlled via the *vanB* operon, was originally described in E. faecalis. In CD630, six putative CTns belonging either to the Tn*916*-like family (CTn*1*, CTn*6*, CTn*7* plus Tn*5397*, previously known as CTn*3*) or to the Tn*1549*-like family (CTn*2*, CTn*4*, and CTn*5*) were identified. Tn*916*-like, CTn*1*-like, and CTn*5*-like were reported from M68, while CTn*5*-like was found in CF5. In this study, CTn*5*-like, Tn*5397*, and Tn*916* elements were identified, with CTn*5*-like found to exist widely among the investigated clade 4 isolates. However, other transposons, which did not belong to either the Tn*916*-like or Tn*1549*-like families, were also discovered, including Tn*4453a/b*-like, Tn*5398*-like, and Tn*6194*-like. Furthermore, two novel putative transposons having regions of homology with CTn*2* and CTn*7* were identified in isolates ZR9 and ZR18, respectively. It is remarkable that in Tn*4453a/b*, *catD* was replaced by *aac(6*′*) aph(2*′′*)* in isolates 2, 7, and 28, resulting in susceptibility to CHL but resistance to gentamicin and amikacin. The three isolates, which were all ST8, were obtained from elderly hospitalized patients treated with quinolone and carbapenem for 5 days. However, isolate ZR18 carried a typical Tn*4453a/b* element with *catD*, leading to CHL resistance. The *aac(6*′*) aph(2*′′*)* gene, displaying 100% identity and coverage with C. jejuni, was first reported in C. difficile. This gene encodes a bifunctional AME, accounting for HLGR resistance rates in >90% E. faecalis and E. faecium isolates (38). This illustrated that HGT may occur between C. difficile and other intestinal bacteria, leading to unique microevolution of individual isolates. However, the mechanisms by which the local microenvironment causes HGT and the ability of Tn*4453a/b* containing *aac(6*′*) aph(2*′′*)* to transfer between C. difficile isolates and/or C. difficile and other bacteria remains to be elucidated. Furthermore, two novel transposons with regions with homology to CTn*2* and CTn*7* were identified. One novel transposon, in isolate ZR9, contained 10 unique genes showing identity with diverse transposons of diverse origins in C. difficile, although the remaining 26 genes were homologous with CTn*2*. The other transposon, in isolate ZR18, lacked 10 out of 24 coding genes present in CTn*7*. The 10 novel genes also displayed identity with C. difficile or other gut pathogens of heterogeneous origins. This situation further suggested that “personalized” HGT occurred in individual isolates, resulting in diversity and genome evolution in C. difficile. Great diversity was also exhibited among the other CTns identified in this study even within the same STs, and several types were classified according to their gene structure and composition.

Sequencing of a number of C. difficile genomes has facilitated the identification of numerous putative prophages by bioinformatic analyses, and the role of prophages in its evolution and virulence has become the focus of recent research. So far, only a limited number of temperate phages infecting C. difficile have been fully characterized in terms of WGS and morphology by transmission electron microscopy (TEM). ΦCD119, ΦC2, ΦCD27, ΦMMP02, and ΦMMP04 are members of the *Myoviridae* family, while ΦCD38-2 and ΦCD6356 are members of the *Siphoviridae* family. In this study, WGS revealed distribution of various numbers of prophages in all isolates. The top seven main prophages in clade 4 were ΦCDHM19, ΦCD506, ΦMMP01, ΦMMP03, ΦMMP02, ΦMMP04, and ΦC2, all of which belong to the *Myoviridae* family. Some prophages (ΦMMP01, ΦMMP03, ΦMMP02, and ΦMMP04) were induced and isolated from fecal supernatant of CDI patients (34). In addition, ΦC2 mediates transduction of Tn*6215*, encoding erythromycin resistance, between C. difficile isolates (11). Our next step is to induce phages of these isolates and confirm their function experimentally.

The CRISPR-Cas systems, which are present in most archaea and many bacteria, provide adaptive resistance against invasive genetic elements, such as phages and plasmids (40, 41). CRISPR array consists of partial palindromic repeats (DR) interspersed by unique DNA sequences (spacers) that are derived from foreign genetic elements in a linear, time-resolved manner (41, 42). Whole-genome analysis of C. difficile strain 630 (ST54/RT012) showed that the CRISPR spacer sequence exhibited similarity to C. difficile phages and plasmids, suggesting CRISPR interference against these mobile elements, which was later experimentally confirmed in the R20291 strain (ST1/RT027/B1) (3, 43). Recently, in a comprehensive analysis of the CRISPR-Cas system in 217 C. difficile genomes, a single type IB CRISPR-*Cas* system and a total of 1,865 CRISPR arrays were identified with *cas* gene clusters present at conserved chromosomal locations (44). The CRISPR arrays of C. difficile were markedly enriched (12.5 arrays/genome) in this study compared with other species and previous reports of C. difficile (8.5 arrays/genome) (45). Numerous CRISPR arrays (90/464) were homologous to prophages identified among the clade 4 C. difficile isolates in this study. CRISPR-Cas has been used for outbreak tracking in Yersinia pestis (46) and Salmonella enterica subspecies (47), and correlating with important phenotypes, such as antibiotic -resistance cassettes in enterococci (48), and prophages in Streptococcus pyogenes genomes (49). All these correlations reflect the role of CRISPR-Cas systems in controlling HGT, as well as the uptake and dissemination of particular genes and operons involved in bacterial adaptation and pathogenesis (50), and hence the evolution of specific species. In our study, we identified another potential method of phylogenetic analysis and genotyping within clade 4 or closely related isolates according to the CRISPR arrays.

Rates of antibiotic resistance vary considerably in different studies, probably depending on the geographic regions and local or national antibiotic policy. In our study, the MDR isolates account for 94.6% of all resistant isolates, which is a much higher proportion than reported previously (55%) in Europe and China (19, 51, 52). Many of the hypervirulent RT027 and RT078 strains are known to exhibit MDR. In a 4-year study in China, MDR isolates with ST37 accounted for the highest proportion (78.2%) of all the tested isolates (53–55). A similar situation was observed in our study, which further illustrates that antibiotic usage promotes independent acquisition of drug resistance, regardless of molecular type.

Resistance to fluoroquinolones has increased dramatically in recent years; for example, the rates of resistance to LVX and CIP reached almost 100% in many studies around the world (51), including our study. Genomic mutations in *gyrA* and/or *gyrB,* known as the quinolone resistance-determining region, QRDR, are involved with resistance to fluoroquinolones (56). The amino acid substitution Thr-82Ile in GyrA was found to be responsible for moxifloxacin resistance (57). Some other substitutions in *gyrA* and/or *gyrB* have been reported in other studies, such as Ser-366Val, Ser-416Ala, Asp-426Asn, and Glu-466Val (58). According to our GWAS analysis, the Thr→IIe mutation in the *gyrA* gene is highly related to MXF resistance in clade 4 C. difficile isolates.

RIF resistance was very recently detected, and studies conducted from 2008 to 2010 in different European countries showed variability in the rates of resistance to this antibiotic in C. difficile strains, ranging from 6.3% to 36.8% (58). In our study, the rate of RIF resistance reached 48.65%, which is even higher than that reported in a systematic meta-analysis of drug resistance of C. difficile infections in mainland China (52). Different SNPs (His-502Asn and Arg-505Lys) within the *rpoB* gene encoding the β-subunit of the RNA polymerase have been identified in RIF-resistant strains (59). Another RIF resistance-related gene, *rphB*, was also detected in 34 isolates in this study but at a low related rate. The existence of some specific substitution in *rphB* that is responsible for RIF remains to be investigated.

In C. difficile, resistance to CLI and ERY is common in Europe (55% and 47%, respectively) (19), and in China (70% to 80%) (60), while in our study the rate was more than 90%. In this study, several genes were identified with *ermB*, high copy numbers of *macB*, and *cfrC*, although the correlation between the phenotype and the harbored gene is low. In pan-Europe studies, the rate of TET resistance has been reported to range from 2.4% to 41.67% (51), and the rate in the present study was slightly lower than the highest level at 35.14%. Moreover, the emergence of reduced TET susceptibility increased in our study. In C. difficile, TET resistance is commonly conferred by a *tet*(M) carried by a Tn*5397* transposon. In our study, 29 isolates contained Tn*5397*; however, *tetM* was absent from isolates ZR73 and 5. Among these 27 isolates, the rate of TET resistance was 40.74% (11/27). For eight isolates without Tn*5397*, two resistant isolates (15 and 16) and one intermediate strain (11032) were still identified. In addition, no other TET resistance-determining genes, including *tet 44* and *tetW* (other important genes responsible for TET resistance in C. difficile especially with animal and environmental origins) were detected in these three isolates. This indicates the possible existence of another mechanism of TET resistance.

Although CHL resistance is not so common in Europe (3.5%) and China (2%) (19, 52), the rate obviously increased in our study (29.73%). It is known that *catD* in Tn*4453a/b* mediates CHL resistance; however, in our study, only one (ZR18) of 11 resistant isolates carried *catD* in Tn*4453a/b* and *cata11* in the genome, which may represent another mechanism of gaining CHL resistance in C. difficile. The great heterogeneity in the genetic arrangement of those resistance determinants confirms that genetic exchange and recombination frequently occur in individual clinical strains.

As reported in a previous study in China, no resistant isolates to MTZ and VAN or isolates with reduced MTZ susceptibility were found here, although reduced MTZ susceptibility was occasionally reported in Europe (61). The mechanism of MTZ resistance is still not completely understood. Nitroimidazole (*nim*) genes conferring resistance to MTZ in Helicobacter pylori have not been identified in C. difficile. The defined mechanism of resistance is a multifactorial process involving alterations in different metabolic pathways, such as nitroreductase activity, iron uptake, and DNA repair (59). A *vanB* operon originally described in E. faecalis harboring Tn*1549* is responsible for resistance to VAN (62). Although several Tn*1549*-like elements have been reported in C. difficile, no functional *vanB* operon was identified. Recently, a *vanG*-like gene cluster, homologous to the cluster found in E. faecalis, has been reported in a number of C. difficile isolates. However, although this cluster is expressed, it does not promote resistance to VAN (63). The same situation was found in isolate ZR18 in our study with a series of *vanG*-like gene clusters identified, without resistance to VAN.

In conclusion, we analyzed HGT-related elements involved in shaping the genome and biological features, including antibiotic susceptibility phenotype of C. difficile from clade 4 with varied STs isolated in China. Our findings provide important new insights into the mechanism of genome remodeling within clade 4 and offer a new method for typing and tracing the origins of closely related isolates.

## MATERIALS AND METHODS

### Isolates and preparation of genomic DNA.

A total of 37 clinical C. difficile isolates from clade 4 with divergent origins in China were included in this study (Table 1). These isolates comprised five ST types: one ST332 isolate (a new type identified in our previous study), four ST 81 (RT 017), one each of ST 39 (RT 085) and ST109, and 30 ST 37 (RT 017) isolates. Details of these isolates are summarized in Table 1. Strains CD630 (GenBank accession no. AM180355), CF5 (FN665652), and M68 (FN668375) were used as references throughout the investigation.

All 37 isolates were cultured on brain heart infusion (BHI) agar plates (Oxoid, Basingstoke, UK) supplemented with 5% sheep blood (BaoTe, Beijing, China) in an anaerobic environment (80% nitrogen, 10% hydrogen, and 10% carbon dioxide) (Mart, NL) at 37°C for 48 h. Typical colonies were picked and recultured on BHI for 24 h before preparation of genomic DNA using the Wizard Genomic DNA purification kit (Promega, Madison, WI, USA) according to the manufacturer’s instructions.

### MLST, PCR ribotyping, and toxin gene profiling.

The MLST, PCR ribotyping, and toxin gene profiling information was obtained according to previously reported methods (37, 64).

### Whole-genome sequencing and assembly.

Whole-genome sequencing (WGS) was performed on the Illumina PE150 (Illumina Inc., USA) with average insert lengths of 350 bp. Raw data were processed in four steps: removal of reads with 5 bp of ambiguous base, reads with 20 bp of low quality (≤Q20), adapter contamination, and duplicated reads. Finally, filtered reads were assembled using SOAP denovo v2.04 (65, 66).

### Genome annotation and phylogenetic analysis.

Genes were predicted using GeneMarkS version 3.05 (67) and aligned with databases to obtain the annotation corresponding to their homologs, with the highest quality alignment result chosen as the gene annotation. Gene functions were predicted by whole-genome BLAST (http://blast.ncbi.nlm.nih.gov/Blast.cgi) searches against the following databases: Gene Ontology (GO) (68), Kyoto Encyclopedia of Genes and Genomes (KEGG) (69), Clusters of Orthologous Groups (COG) (70), Non-Redundant Protein (NR) databases (71), Transporter Classification Database (TCDB) (72), and Swiss-Prot (73). tRNA genes were predicted by tRNA scan-SE (74). rRNA genes were analyzed by rRNAmmer (75). Small nuclear RNAs (snRNA) were predicted by BLAST searches against the Rfam (76) database.

Single core genes were obtained from the clustering results of all sample genes used by CD-HIT version 4.6. A phylogenetic tree was built using the neighbor-joining method based on 2,456 single core genes using MEGA6.0 with 1,000 replicates (77).

### Analysis of MGEs.

All the transposons (Tns) and conjugative transposons (CTns) were identified using BLAST searches of the C. difficile genome sequences or transposon sequences available at NCBI ( https://www.ncbi.nlm.nih.gov/) with identity and coverage requirements set at >80%. Prophages were identified using the PHASTER web server (http://phast.wishartlab.com). The intact, questionable, and incomplete prophage sequences were defined by score values of >90, 70 to 90, and <70, respectively (78). To identify plasmids, reads were assembled into contigs using the SOAP denovo. Contigs were then screened for plasmids using Microbial Genome BLAST searches against the NCBI complete plasmid database (ftp://ftp.ncbi.nlm.nih.gov/genomes/refseq/plasmid/). The potential plasmids were defined as ≥70% coverage and ≥80% identity. The CRISPRFinder (79) was used for CRISPR identification.

### Sequence analysis of PaLoc and CdtLoc.

The PaLoc and CdtLoc were confirmed by comparison with the reference CD630 genome. Orthologous genes were detected by BLAST (version 2.2.12) searches with >80% coverage and >90% nucleotide identity. The genetic structure as well as insertions and deletions (indels) were studied.

### Antimicrobial susceptibility tests.

*C. difficile* isolates were tested for susceptibility to moxifloxacin (MXF), vancomycin (VAN), clindamycin (CLI), tetracycline (TET), erythromycin (ERY), rifampin (RIF), levofloxacin (LVX), chloramphenicol (CHL), metronidazole (MTZ), ciprofloxacin (CIP), and meropenem (MEM) using Etest strips (bioMérieux, France, and Liofilchem, Italy). Tests to gentamicin (CEN) and amikacin (AMK) were also performed with isolates 2, 7, 28, and ZR18 using Etest strips (bioMérieux, France, and Liofilchem, Italy). The MICs for MTZ, MXF, CLI, CIP, LVX, and TET were determined according to recommendations of the Clinical and Laboratory Standards Institute (CLSI) M11-A7 and M100-S24 (80, 81), and the European Committee on Antimicrobial Susceptibility Testing (EUCAST) (http://www.eucast.org). The breakpoints for VAN, RIF, ERY, CHL, and meropenem were determined according to a previous study (54, 58). Multidrug resistance (MDR) was defined as resistance to at least three antimicrobial classes. C. difficile ATCC 700057 was included as a control in each experiment (82).

Antimicrobial resistance genes were predicted through comparison with the Antibiotic Resistance Genes Database, ARDB https://ardb.cbcb.umd.edu/ (35), and the Comprehensive Antibiotic Resistance Database, CARD https://card.mcmaster.ca/ (36). Heatmap analysis was performed using the pheatmap package, and statistics packages in R software (version 2.15.3).

### Single nucleotide polymorphisms detected and association analysis.

Single nucleotide polymorphisms (SNPs) were detected by comparing the sequence of each isolate with that of strain BJ08. SNP calling was detected using the NUCmer program in the MUMer package (V3.23). All primary SNPs were further evaluated in contigs to validate alleles. Sequence regions of 201 bp, which included 100 bp extracted from either side of the referenced SNP site, were subjected to BLAST analysis with sample contigs. The SNPs were removed if the alignment length was less than 101 bp. In addition, the SNPs located in repeat regions predicted by RepeatMasker (version open-4.05) and TRF (Tandem Repeats Finder; version 4.07b) of the reference were also excluded. Genome-wide association studies (GWAS) were conducted using the open-source R package treeWAS, which is freely available at https://github.com/caitiecollins/treeWAS (83). Three methods (terminal, simultaneous, and subsequent tests) were used to confirm the results in this study.

### Data availability.

The sequence information from this whole-genome shotgun project of 37 C. difficile isolates has been submitted to DDBJ/EMBL/GenBank under the following BioProject accession number PRJNA479396.
