# Supplementary material for: Independent Microevolution Mediated by Mobile Genetic Elements of Individual Clostridium difficile Isolates from Clade 4 Revealed by Whole-Genome Sequencing
Source: mSystems. 2019 Mar 26;4(2):e00252-18. doi: 10.1128/mSystems.00252-18 (PMC6435816; doi:10.1128/mSystems.00252-18)
Supplement: TABLE S1 [file mSystems.00252-18-st001.pdf]

**Table 1.** Common genomic features of 37 *C. difficile* isolates

| Isolate | Genome size (bp) | GC% | Contigs | Coding sequence | tRNA |
|---|---|---|---|---|---|
| 2 | 4244657 | 28.5 | 67 | 4021 | 72 |
| 4 | 4232675 | 28.51 | 65 | 3981 | 72 |
| 5 | 4213976 | 28.57 | 312 | 3933 | 27 |
| 6 | 4137064 | 28.52 | 80 | 3843 | 73 |
| 7 | 4251250 | 28.49 | 67 | 4048 | 77 |
| 15 | 3991878 | 28.8 | 1366 | 3363 | 13 |
| 16 | 4214708 | 28.61 | 69 | 3973 | 72 |
| 23 | 4251260 | 28.47 | 62 | 3997 | 71 |
| 28 | 4259545 | 28.63 | 53 | 4020 | 75 |
| 29 | 4148034 | 28.5 | 72 | 3859 | 69 |
| 35 | 4104332 | 28.31 | 94 | 3816 | 43 |
| 38 | 4215490 | 28.52 | 71 | 3958 | 55 |
| 50 | 4099906 | 28.55 | 62 | 3773 | 55 |
| BJ08-1 | 4237586 | 28.62 | 43 | 3993 | 46 |
| GZ11 | 4229048 | 28.54 | 68 | 3955 | 75 |
| GZ12 | 4257298 | 28.65 | 69 | 3986 | 72 |
| GZ13 | 4304711 | 28.68 | 66 | 4093 | 75 |
| GZ14 | 4333573 | 28.8 | 92 | 4101 | 72 |
| GZ2 | 4225872 | 28.52 | 62 | 3957 | 78 |
| GZ3 | 4267686 | 28.74 | 67 | 3984 | 79 |
| GZ6 | 4210800 | 28.47 | 62 | 3945 | 58 |
| GZ8 | 4197923 | 28.46 | 258 | 3898 | 12 |
| HN9 | 4224838 | 28.6 | 190 | 3962 | 12 |
| ZR18 | 4120911 | 28.84 | 531 | 3721 | 30 |
| ZR29 | 4300339 | 28.87 | 75 | 4060 | 66 |
| ZR58 | 4320956 | 28.89 | 60 | 4107 | 59 |
| ZR59 | 4323199 | 28.91 | 96 | 4094 | 72 |
| ZR65 | 4223364 | 28.51 | 81 | 4020 | 28 |
| ZR66 | 4315448 | 28.88 | 84 | 4078 | 59 |
| ZR68 | 4229879 | 28.5 | 70 | 4026 | 34 |
| ZR72 | 4285694 | 28.62 | 76 | 4058 | 72 |
| ZR73 | 4271512 | 28.61 | 219 | 4035 | 45 |
| ZR8 | 4180553 | 28.52 | 79 | 3873 | 70 |
| ZR82 | 4269495 | 28.58 | 111 | 4064 | 24 |
| ZR9 | 4231111 | 28.66 | 171 | 3915 | 75 |
| 10122 | 4167226 | 28.65 | 76 | 3875 | 64 |
| 11032 | 4090702 | 28.5 | 177 | 3767 | 33 |
