# Supplementary material for: Independent Microevolution Mediated by Mobile Genetic Elements of Individual Clostridium difficile Isolates from Clade 4 Revealed by Whole-Genome Sequencing
Source: mSystems. 2019 Mar 26;4(2):e00252-18. doi: 10.1128/mSystems.00252-18 (PMC6435816; doi:10.1128/mSystems.00252-18)
Supplement: TABLE S4 [file mSystems.00252-18-st004.pdf]

S Table 4 Potential plasmids sequences identified

Plasmid	ID	Strain	Organism	Size (bp)	Gene	Isolates dispersed
pSK1	NG_047055	SK18	*S. aureus*	1,640	*AAC(6')-APH(2")*	15,16,2.28,38,5,7,BJ08,GZ13,GZ14,HN9,ZR18,ZR29, ZR58, ZR59,ZR65,ZR66,ZR68,ZR72,ZR73,ZR82, 10122, 11032
pGTK3	NG_047212	No.3	*S. warnei*	1,640	*AAC(6')-APH(2")*	Same as above
pMCCL2	NG_047217	JCSC5402	*M.caseolyticus*	1,676	*AAC(6')-APH(2")*	Same as above
pCG8245	NG_047405	CG8245	*C. jejuni*	1,094	*AAC(6')-APH(2")*	15,2,28,7, 10122, 11032
pEOC01	NG_047378	NCIMB 6990	*P. acidilactici*	994	*aadE*	16,38,5,BJ08,GZ11,GZ13,GZ14,GZ2,HN9,ZR18,ZR29,ZR58,ZR59,ZR65 ,ZR66,ZR68,ZR72,ZR73,ZR82, 11032
pAM-beta1	NG_047794	AM-beta1	*E. faecalis*	938	*erm(B)*	16,2,28,29,38,4,5,6,7,GZ11,GZ12,GZ13,GZ14,GZ2,GZ3,GZ6,GZ8,ZR29, ZR58,ZR59,ZR66,ZR8,ZR9, 10122
pBT233	NG_047797		*S.pyogenes**	938	*erm(B)*	Same as above
pLEM89	NG_047799	LEM89	*L. fermentum*	953	*erm(B)*	Same as above
pLUL631	NG_047803		*L. reuteri*	938	*erm(B)*	Same as above
pTE15	NG_047842	N16	*L. reuteri*	947	*erm(B)*	Same as above
pMD5057	NG_048225	5057	*L. plantarum*	2.120	*tet(M)*	2,23,28,29,38,4,5,6,7,BJ08,GZ11,GZ12,GZ13,GZ14,GZ2,GZ3,GZ6,GZ8, HN9, ZR18,ZR29,ZR58, ZR59, ZR66, ZR72,ZR73,ZR82, ZR8,ZR9, 10122
pYA470-4	NG_048233	9830470-4	*E. faecium*	1,920	*tet(M)*	Same as above
pK214	NG_048274	K214	*L. lactis**	2,141	*tet(S)*	Same as above, except for ZR18
pKL0018	NG_048277	KL0018	*L*. garvieae*	2,141	*tet(S)*	Same as above, except for ZR18
pCC31	NG_048259		*C. coli*	2,020	*tet(O)*	ZR18, 10122
pCG8245	NG_047322	CG8245	*C. jejuni*	977	*aad9*	11032

M is for Macrococcus; C is for Campylobacter; P is for Pediococcus; S* is for Streptococcus; L is for Lactobacillus; L* is for Lactococcus;
